# Sex-dependent bioactive lipid mediator profiles in multiple sclerosis

**DOI:** 10.1177/20552173261475501

**Published:** 2026-08-08

**Authors:** Wing Hee Fung, Serhii Chornyi, Menno M Schoonheim, Bernard MJ Uitdehaag, Joep Killestein, Eva MM Strijbis, Gijs Kooij

**Affiliations:** 1 MS Center Amsterdam, Neurology, 1209 Vrije Universiteit Amsterdam, Amsterdam Neuroscience, Amsterdam UMC, Location VUmc, Amsterdam, The Netherlands; 2 MS Center Amsterdam, Molecular Cell Biology and Immunology, Vrije Universiteit Amsterdam, Amsterdam Neuroscience, 522567 Amsterdam UMC, Location VUmc, Amsterdam, The Netherlands; 3 MS Center Amsterdam, Anatomy and Neurosciences, 1209 Vrije Universiteit Amsterdam, Amsterdam Neuroscience, Amsterdam UMC, Location VUmc, Amsterdam, The Netherlands

**Keywords:** Multiple sclerosis, lipid mediators, expanded disability status scale, neurofilament, 9-HOTrE

## Abstract

**Background:**

Pathophysiological drivers of sex differences in multiple sclerosis (MS) remain unclear. Potential candidates include bioactive lipid mediators (LMs), which are pivotal regulators of neuro-inflammatory responses.

**Objective:**

To investigate sex-dependent LM differences in MS.

**Methods:**

Plasma lipids were measured using targeted HPLC-MS/MS in a birth-year MS cohort (N = 410; n = 285 MS, n = 169 RRMS, n = 80 SPMS and n = 36 PPMS). Lipid profiles were compared between sexes and correlated with disability (Expanded Disability Status Scale (EDSS)), neuroaxonal damage (neurofilament light, NfL), whole brain volume (WBV) and T2 lesion volume (LV).

**Results:**

In healthy controls, 9-HOTrE levels were higher in women than men. In individuals with MS, women had higher 9-HOTrE, 18-HEPE and 9-HODE levels than men, while men had higher 15-HEPE, 17-HDHA and 11,12-DiHET levels than women. Among these LMs, increased 11,12-DiHET levels were associated with higher EDSS and NfL levels, while higher levels of the remaining LMs, such as 9-HOTrE, were associated with lower EDSS and lower LV.

**Conclusion:**

These findings provide insights into potential sex-dependent lipid-mediated pathophysiological mechanisms in MS patients. Our findings suggest that certain LMs, such as 9-HOTrE, may display neuroprotective effects in MS.

## Introduction

Phenotypic variability exists between men and women with multiple sclerosis (MS), with men more likely to experience a progressive disease course compared to women.^1^ In addition, the female-to-male ratio of having MS is approximately 3:1.^2^ The mechanisms behind these differences are unclear, but sex-specific effects on the onset and resolution of inflammatory responses, mediated by bioactive lipid mediators (LMs) derived from omega-3 and omega-6 polyunsaturated fatty acids, have been suggested. For example, a distinct LM profile has been observed between men and women in neurological diseases other than MS, such as cerebrovascular disease.^3,4^ The chronic and persistent neuro-inflammatory phenotype of MS is thought to result partly from a dysregulated resolution response, driven by an imbalance of pro-inflammatory and pro-resolving LMs.^5^ Especially, our group has shown that the AA pathway is predominantly involved in MS.^6^ During the acute phase of inflammation, generally AA-derived eicosanoids like prostaglandins and leukotrienes are biosynthesized.^7^ Such bioactive trigger responses like vasoconstriction, changes in vascular permeability, vasodilation and oedema. Interestingly, prostaglandins like PGE_2_ and PGD_2_ also help to stop inflammation by promoting the activation of 15-LOX, leading to the transition from eicosanoid biosynthesis to lipoxin formation, a process known as LM class switching.^8,9^ Unlike rapidly produced prostaglandins and leukotrienes, lipoxins appear later and are associated with the resolution phase of inflammation. Studying sex-dependent differences in MS is therefore relevant given the differences in susceptibility, disease progression and general immune responses between men and women.^10^ We here examined sex-dependent differences in plasma LM profiles in a birth-year MS cohort of MS patients born in 1966,^11^ thereby controlling for age as a confounding factor. We also correlated observed LM differences with clinical (Expanded Disability Status Scale, EDSS), biochemical (neurofilament light, NfL), and MRI parameters such as whole brain volume (WBV) and T2 lesion volume (LV).

## Methods

### Cohort description

Patients with MS were selected from a population-based cross-sectional birth year cohort, referred to as Project Y.^11^ Project Y aimed to recruit all Dutch individuals born in 1966 and diagnosed with MS according to the 2017 McDonald criteria, thereby minimizing age-related confounding. A comprehensive interview including MS history and general medical history was conducted. With the patient's permission, all available medical documentation was subsequently reviewed to determine, among other factors, the MS subtype.^11^ In project Y, age- and sex-matched healthy controls (HC) were also included. Participants were evaluated during a 1-day visit at Amsterdam University Medical Center, between December 2017 and January 2021. Participants had a comprehensive examination consisting, amongst others, of performance tests, EDSS, and an MRI scan. Blood plasma was collected for biobank storage and analysis.

### Lipid mediator analysis

We measured lipids in plasma using a targeted high-performance liquid chromatography-tandem mass spectrometry (HPLC-MS/MS).^6^ The relative abundance of lipids was defined as relative to the corresponding internal standards.^6^ Specific lipids were excluded from the dataset if the percentage of samples with values below the detection limit was above 20% in all groups (men MS, men HC, women MS, women HC). We then imputed remaining missing values with the Gibbs sampler-based left-censored missing value imputation approach (GSimp).^12^ After the imputation, 35 lipids were used for further analysis. These lipids are also represented in Table S1.

### Neurofilament light, whole brain and lesion volume (WBV and LV) measurements

NfL was assessed in serum using a single-molecule array assay on a HD-X analyzer as described previously.^13^ Image acquisition and image processing for WBV and LV have been described previously.^14^ In short, MRI was performed at 3T on a single scanner for all participants using standardized protocols across the entire cohort. Brain imaging included 3D T1-weighted sequences for volumetric analyses and 3D FLAIR sequences for lesion detection. White matter hyperintensities were semi-automatically segmented on FLAIR images using a validated neural network approach, followed by manual quality control. Total T2-hyperintense lesion volume was derived from these masks, and gadolinium-enhancing lesions were not included in this measure. Lesion-filled T1 images were subsequently generated for whole-brain volumetric analysis. Brain volumes were quantified using established FSL-based tools, including SIENAX for estimation of total brain volume with head-size normalization.

### Statistical analysis

First, we compared lipid levels between men and women in HC and MS. Differentially expressed lipids between men and women with MS were correlated with EDSS, NfL and WBV in the entire MS group, and within each sex separately using partial Spearman correlation analyses, adjusted for disease-modifying therapy, disease duration, MS subtype (RRMS/PMS) and BMI (only for NfL correlations). Group comparisons of individual lipids were performed using two-sample t-tests across the following comparisons: women versus men in HC, women versus men in MS, HC versus MS in men, HC versus MS in women, women versus men in RRMS, and women versus men in PMS. *P* < 0.05 was considered significant after Benjamini-Hochberg correction for all analyses. We conducted statistical analyses using R (version 4.3.2).

## Results

### Patient characteristics

**Figure 1.**
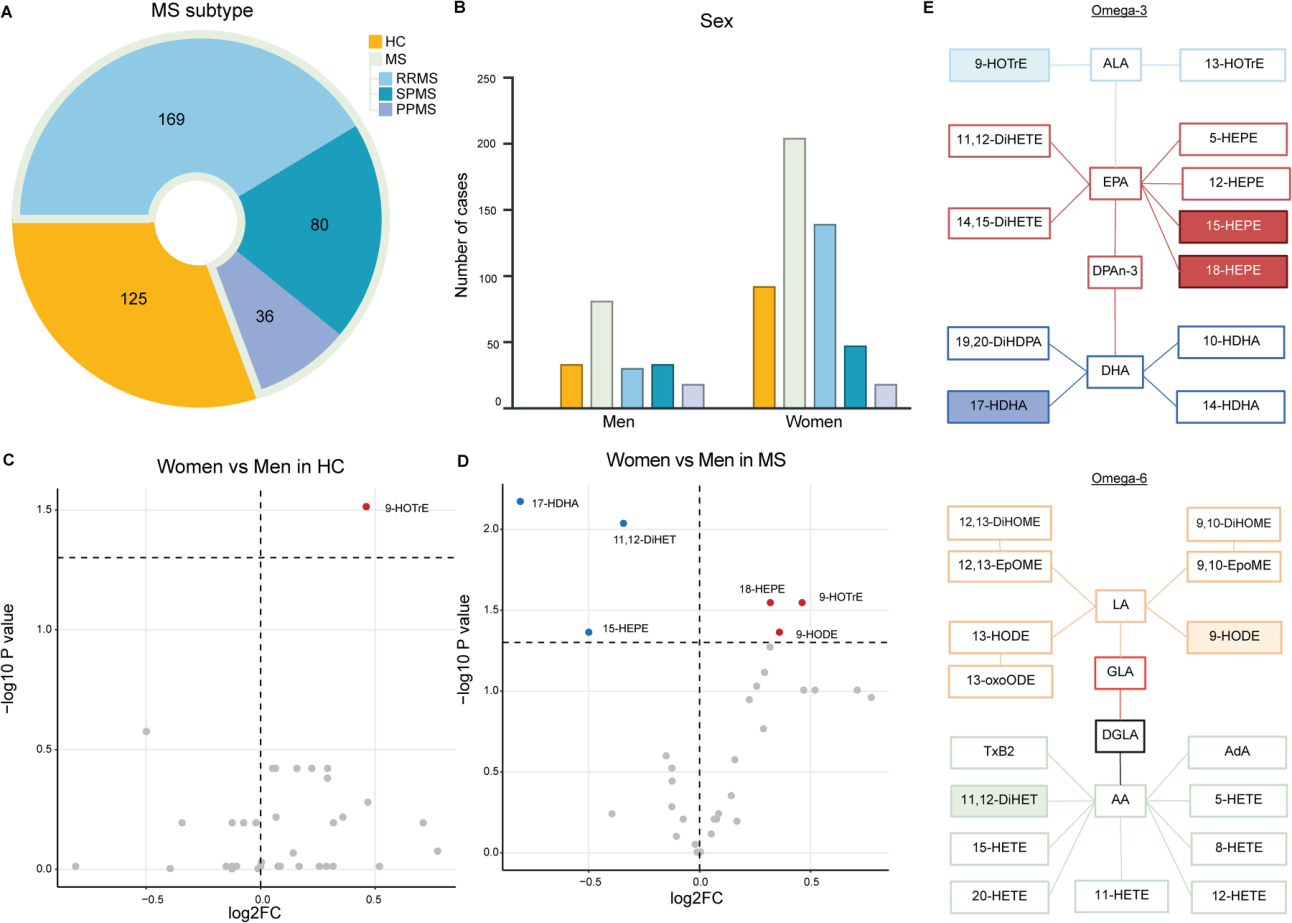
**(**A, B) Cohort demographics (MS subtype and sex). (C, D) Volcano plot showing the log2 fold difference of lipid mediator levels between men and women in MS or in healthy controls (HC). Level of significance (*P* < 0.05) is indicated on the *y*-axis as –log10(*p*-value), adjusted for multiple testing. (E) Lipid mediators derived from omega-3 and omega-6 polyunsaturated fatty acids. All identified lipid mediators from the analyses are presented. Filled coloured boxes indicate lipid mediators with significantly different expression levels between men and women with MS.

Of the 367 patients with MS and 125 HCs who participated in the Project Y cohort, a total of 285 patients with MS (169 patients with RRMS, 80 with SPMS and 36 with PPMS) and 125 age-matched HCs had available plasma samples and were selected for this study (Figure 1(A) and (B)). RRMS was more common in women (68.2%) than in men (37.0%), whereas men were overrepresented in both SPMS (40.7% in men vs 23.0% in women) and PPMS (22.2% in men vs 8.8% in women). EDSS was significantly higher in men (3.5 [0.0–4.4]) than in women (2.5 [0.0–4.0]), and there was no difference in time since last relapse between men and women. Cohort demographic details are provided in Table S2, stratified per MS subtype (Tables S3–S5). A difference in treatment distribution was observed only in the PPMS subgroup, whereas no differences were found in the RRMS and SPMS subgroups or in the overall cohort.

### Different plasma levels of LMs between men versus women in HC and MS

In HC, only alpha-lipoic acid (ALA)-derived 9-HOTrE levels were significantly higher in women compared to men (Figure 1(C)). In MS, this LM was also found to be higher in women than in men. In addition, women with MS also had higher levels of eicosapentaenoic acid (EPA)-derived 18-HEPE and linolenic acid (LA)-derived 9-HODE compared to men with MS. In contrast, women displayed lower levels of EPA-derived 15-HEPE, docosahexaenoic acid (DHA)-derived 17-HDHA, and arachidonic acid (AA)-derived 11,12-DiHET compared to men (Figure 1(D)). An overview of altered LM levels from both the omega-3 and omega-6 pathways is highlighted in Figure 1(E). To assess whether these differences were not driven by the frequency of SPMS and PPMS in men, we next compared the lipid levels between women and men separately in RRMS and PMS. No differences in lipid levels were found between men and women in RRMS; however, in PMS, women with MS had lower levels of 17-HDHA compared to men with MS (Figure S1A-B).

### Different plasma levels of LMs between HC vs MS in men and women

When comparing the LMs levels between HC and MS in men, we found that 9-HODE, 9-HOTrE, 13-HOTrE and LA-derived 12,13-EpOME levels were higher in HC than in MS, whereas no significant differences were observed between HC and MS in women (Figure S1C-D). When comparing HC versus MS, without stratifying by sex, no differences in lipids were found (data not shown).

### Sex-dependent correlations between LMs, clinical, biochemical and MRI measures

**Figure 2.**
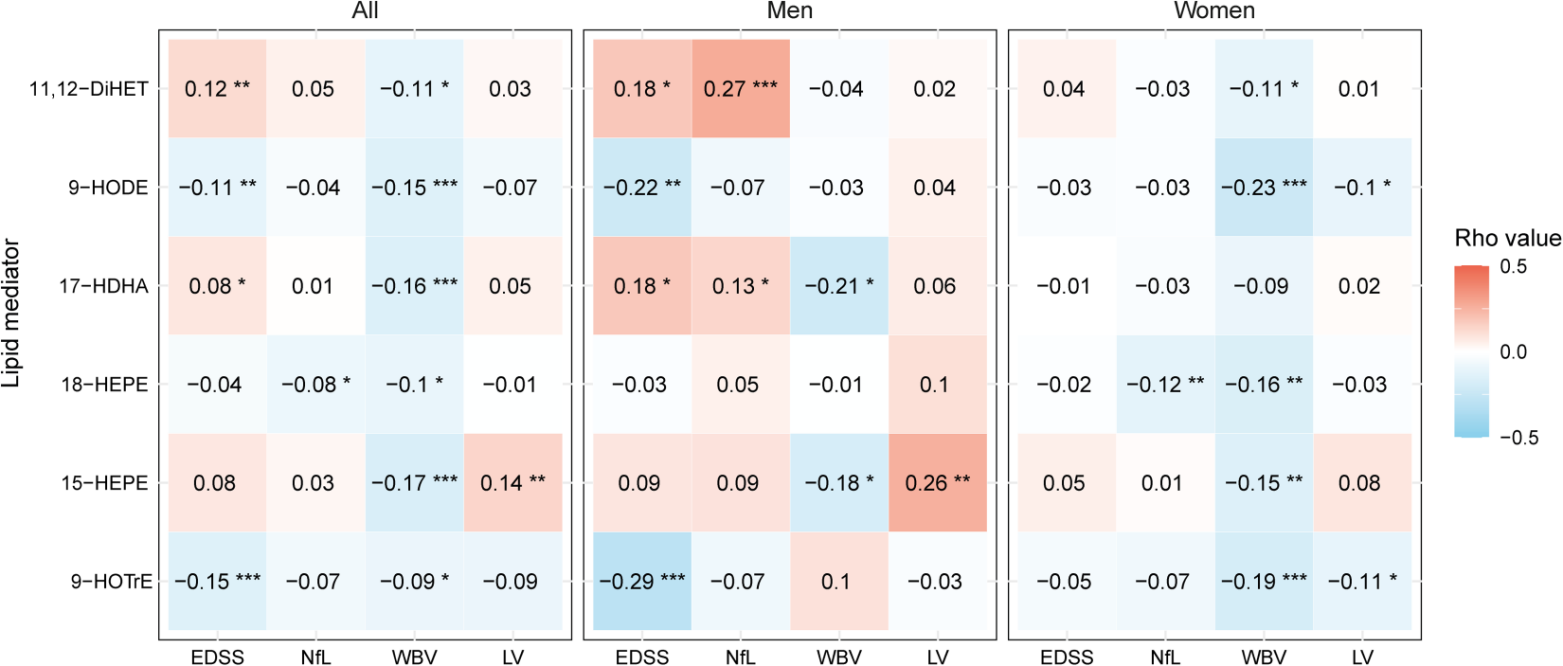
Partial Spearman correlation analysis between differentially expressed lipid mediators and Expanded Disability Status Scale (EDSS), neurofilament light (NfL), whole brain volume (WBV) and lesion volume (LV), corrected for disease-modifying therapy use, disease duration, MS subtype (RRMS/PMS) and body mass index (only for NfL correlations). Correlations are done in the overall MS cohort and stratified by sex. * *P* < 0.05, ** *P* < 0.01, *** *P* < 0.001, adjusted for multiple testing.

To investigate whether the differentially expressed LMs between men and women with MS were associated with clinical, biochemical and MRI parameters, we next correlated specific LM levels with EDSS, NfL, WBV and LV in the whole MS group, and separately in men and women with MS (Figure 2). We observed a weak negative correlation of EDSS with 9-HOTrE and 9-HODE in men but not in women; for NfL, a weak positive correlation was observed with 11,12-DiHET in men but not in women; for WBV, a weak negative correlation with 17-HDHA in men, and a weak negative correlation with 9-HOTrE, 9-HODE, 18-HEPE and 15-HEPE in women (Figure 2 and Figure S2–S4). Lastly, a weak positive correlation between LV and 15-HEPE was seen in men, and a weak negative correlation between LV and 9-HODE and 9-HOTrE was seen in women. Taken together, the LMs that differed between males and females with MS also show sex-dependent correlations with EDSS, NfL, WBV and LV. Notably, higher levels of 11,12-DiHET showed the strongest correlation with increased NfL levels in men, but not in women. Additionally, 9-HOTrE was the only LM that showed a gender-specific difference in HC that correlated negatively with EDSS and LV.

## Discussion

Pathophysiological mechanisms underlying sex differences in MS remain poorly understood. Potential contributors include bioactive LMs derived from omega-3 and omega-6 PUFA, which play a role in the onset and resolution of inflammatory responses. In a population-based birth cohort study, we found six LMs that were different between men and women with MS. These lipids also showed sex-dependent correlations with clinical, biochemical and MRI parameters like EDSS, NfL, WBV, and/or LV.

In HC, 9-HOTrE was the only LM that showed a significant sex difference, with higher levels in women than men. While there is limited research focusing on LM profiles in HCs between sexes, a study examining urine reported similar findings, with 9-HOTrE levels approximately two-fold higher in women compared to men.^15^ In our study, this sex difference in 9-HOTrE levels was also observed in individuals with MS. Correlation analyses revealed that higher 9-HOTrE levels were associated with lower EDSS across the entire MS cohort. This negative correlation was also present in men with MS but not in women with MS, which may be explained by the generally lower average EDSS scores observed in women compared to men. In addition, higher 9-HOTrE levels were also correlated with lower LV in women. These findings suggest a potential neuroinflammatory protective role for 9-HOTrE in MS, given its elevated levels in healthy women and its association with LV in MS, which is also reflected in reduced disability. However, since higher 9-HOTrE levels were also correlated with lower WBV, it is possible that while 9-HOTrE may exert anti-inflammatory effects, it may not confer protection against neurodegeneration.

Sex-specific differences in LMs beyond 9-HOTrE were observed exclusively in individuals with MS and not in HC. These findings suggest that the altered lipid profiles represent sex-dependent pathophysiological responses specific to MS. To date, only a few studies have investigated putative sex differences in peripheral LM profiles in MS. One study reported higher levels of AA-derived 11,12-DiHET in men compared to women, consistent with our observations.^16^ As DiHETs are metabolites of EETs via soluble epoxide hydrolase (sEH), their elevated levels in men may indicate increased sEH expression and/or activity. The enzyme sEH plays a crucial role in the metabolism of epoxy fatty acid mediators and has emerging significance in regulating inflammation.^17^ Also, pharmacological sEH inhibition has been shown to reduce inflammatory macrophage activation.^18^ Increased sEH expression has been linked to worse clinical outcomes, for example in ischemic brain injury.^19^ This suggests that lower DiHET levels in women with MS may reflect differences in sEH activity, potentially indicating a distinct inflammatory environment in men and women. However, given the limited and exploratory nature of the current evidence, these findings should be interpreted with caution and validated in independent cohorts.

In addition, we found that women with MS had higher levels of LA-derived 9-HODE and ALA-derived 9-HOTrE. While sEH primarily acts within the AA pathway, sEH inhibition also affects other lipid pathways, including LA and ALA, with greater LM modifications in men. Specifically, sEH inhibition has been linked to lower 9-HOTrE and 9-HODE levels in men but not in women.^20^ In our study, higher levels of 9-HOTrE and 9-HODE correlated with lower EDSS in men, but these correlations were not observed in women. These findings suggest potential sex-dependent regulation of LM biosynthesizing enzymes, like sEH, and subsequent inflammatory responses in MS.

Furthermore, the EPA-derived 15-HEPE and DHA-derived 17-HDHA were found to be lower in women than in men with MS. Both LMs are biosynthesized via ALOX15/ALOX15B enzymes, as shown in macrophages.^21^ In earlier research, ALOX15/ALOX15B levels were consistently found in all MS types; however, no differences were made between men and women.^5^ Previous research during severe lung infections revealed that the expression of ALOX15/ALOX15B was significantly elevated in males, but its expression in women remains inconclusive.^22^ Given the pivotal role of ALOX15 in neurodegenerative processes,^6,23^ the observed correlation between higher 15-HEPE levels and lower WBV in women, but not in men, warrants further investigation.

While previous studies have compared LM profiles between individuals with MS and HCs, they have not specifically addressed sex-based differences in LMs.^5,6^ We observed generally weak correlations between LMs and clinical, biochemical and MRI parameters; however, clear differences in peripheral LM levels were observed between men and women. A limitation of this study is that hormone differences between men and women, such as oestrogen, can also cause LM differences.^24^ On the other hand, all participants in this study were born in the same year, thereby effectively controlling for age as a confounding factor, since it is known that plasma LM can vary with age.^25^ However, in the present study, the sample size appears insufficient to support robust clinical conclusions, and key potential confounders such as comorbidities, lifestyle factors and dietary habits that may influence lipid levels were not fully accounted for. Future studies combining plasma and cerebrospinal fluid lipids may further link sex differences to local and systemic LM alterations. Given the role of lipid-biosynthesizing enzymes in modulating LM levels, future research should also investigate their cellular expression and enzymatic activity in MS in men and women, as targeting these pathways may have differential therapeutic effects based on sex.

In conclusion, in a cohort of people with MS of the same age, we observed distinct LM profiles between men and women. These LMs also displayed some sex-dependent correlations with EDSS, NfL, WBV and/or LV. Our findings suggest that LM differences potentially reflect underlying lipid-mediated pathophysiological mechanisms that may contribute to sex-specific disease processes in MS. Based on the data, some LMs may have a sex-specific neuroprotective role, such as 9-HOTrE; however, these findings should be interpreted as exploratory and further research is needed to confirm these observations.

## Supplemental Material

sj-docx-1-mso-10.1177_20552173261475501 - Supplemental material for Sex-dependent bioactive lipid mediator profiles in multiple sclerosis Supplemental material, sj-docx-1-mso-10.1177_20552173261475501 for Sex-dependent bioactive lipid mediator profiles in multiple sclerosis by Wing Hee Fung, Serhii Chornyi, Menno M Schoonheim, Bernard MJ Uitdehaag, Joep Killestein, Eva MM Strijbis and Gijs Kooij in Multiple Sclerosis Journal – Experimental, Translational and Clinical
