# Supplementary material for: Sex-dependent bioactive lipid mediator profiles in multiple sclerosis
Source: Mult Scler J Exp Transl Clin. 2026 Aug 8;12(3):20552173261475501. doi: 10.1177/20552173261475501 (PMC13452923; doi:10.1177/20552173261475501)
Supplement: Supplementary file 1 [file sj-docx-1-mso-10.1177_20552173261475501.docx]

**Supplementary material**

| **Table S1. Lipids included in the analysis** | | | | | | | | |
| --- | --- | --- | --- | --- | --- | --- | --- | --- |
| Omega-3 | | | | Omega-6 | | | | |
| ALA | EPA | DPAn-3 | DHA | LA | GLA | DGLA | AdA | AA |
| 9-HOTrE | 11,12-DiHETE |  | 19,20-DiHDPA | 9,10-DiHOME |  |  |  | TxB2 |
| 13-HOTrE | 14,15-DiHETE |  | 10-HDHA | 9,10-EpOME |  |  |  | 11,12-DiHET |
|  | 5-HEPE |  | 14-HDHA | 12,13-DiHOME |  |  |  | 5-HETE |
|  | 12-HEPE |  | 17-HDHA | 12,13-EpOME |  |  |  | 8-HETE |
|  | 15-HEPE |  |  | 9-HODE |  |  |  | 11-HETE |
|  | 18-HEPE |  |  | 13-HODE |  |  |  | 12-HETE |
|  |  |  |  | 13-oxoODE |  |  |  | 15-HETE |
|  |  |  |  |  |  |  |  | 20-HETE |
| Overview of lipids included in the analysis, divided in omega-3 and omega-6 lipids with the polyunsaturated fatty acids and derived lipid mediators. | | | | | | | | |

| **Table S2. Demographics** | | | |
| --- | --- | --- | --- |
|  | **MS (N=285)** | | |
|  | **Men (n=81)** | **Women (n=204)** | **p value** |
| **MS subtype, n (%)** |  |  | <0.001 |
| RRMS | 30 (37.0%) | 139 (68.2%) |  |
| SPMS | 33 (40.7%) | 47 (23.0%) |  |
| PPMS | 18 (22.2%) | 18 (8.8%) |  |
| **Disease duration** | 11.6 (0.0-19.1) | 9.0 (0.0-20.4) | 0.68 |
| **Time since last relapse, weeks** | 66.0 (26.8-98.3) | 58.5 (25.0-109.8) | 0.91 |
| **Disease-modifying therapy, n (%)** |  |  | 0.23 |
| Dimethyl fumarate | 11 (13.6%) | 25 (12.3%) |  |
| Interferon-beta | 18 (22.2%) | 27 (13.2%) |  |
| Glatiramer acetate | 5 (6.2%) | 16 (7.8%) |  |
| Natalizumab | 1 (1.2%) | 10 (4.9%) |  |
| Cladribine | 1 (1.2%) | 3 (1.5%) |  |
| Fingolimod | 6 (7.4%) | 10 (4.9%) |  |
| Alemtuzumab | 1 (1.2%) | 0 (0.0%) |  |
| None | 38 (46.9%) | 113 (55.4%) |  |
| **EDSS** | 3.5 (0.0-4.4) | 2.5 (0.0-4.0) | 0.02 |
| **NfL** | 9.4 (7.6-11.2) | 9.6 (7.5-11.7) | 0.77 |
| **Mean whole brain volume (L), SD** | 1.47 (0.08) | 1.49 (0.07) | 0.046 |
| **T2 lesion volume (mL)** | 10.82 (6.0-11.5) | 10.79 (6.0-17.6) | 0.42 |
| P values from χ2 test, Mann-Whitney test for median, and two sample t-test for mean. Data presented as n (%) or median (IQR), unless otherwise indicated. | | | |

| **Table S3. Demographics of the patients with RRMS** | | | |
| --- | --- | --- | --- |
|  | **RRMS (N=169)** | | |
|  | **Men (n=30)** | **Women (n=139)** | **p value** |
| **Disease duration** | 13.4 (9.3-18.6) | 14.5 (8.1-25.3) | 0.38 |
| **Time since last relapse, weeks** | 60.0 (17.5-81.5) | 58.5 (20.3-115.0) | 0.54 |
| **Disease-modifying therapy, n (%)** |  |  | 0.20 |
| Dimethyl fumarate | 6 (20.0%) | 20 (14.4%) |  |
| Interferon-beta | 7 (23.3%) | 19 (13.7%) |  |
| Glatiramer acetate | 3 (10.0%) | 15 (10.8%) |  |
| Natalizumab | 1 (3.3%) | 8 (5.8%) |  |
| Cladribine | 0 (0.0%) | 2 (1.4%) |  |
| Fingolimod | 3 (10.0%) | 8 (5.8%) |  |
| Alemtuzumab | 1 (3.3%) | 0 (0.0%) |  |
| None | 9 (30.0%) | 67 (48.2%) |  |
| **EDSS** | 3.3 (2.5-4.0) | 3.0 (2.0-4.0) | 0.37 |
| **NfL** | 9.2 (7.6-11.0) | 9.3 (7.4-11.5) | 0.88 |
| **Mean whole brain volume (L), SD** |  |  | 0.13 |
| **T2 lesion volume (mL)** |  |  | 0.82 |
| P values from χ2 test, Mann-Whitney test for median, and two sample t-test for mean. Data presented as n (%) or median (IQR), unless otherwise indicated. | | | |

| **Table S4. Demographics of the patients with SPMS** | | | |
| --- | --- | --- | --- |
|  | **SPMS (N=80)** | | |
|  | **Men (n=33)** | **Women (n=47)** | **p value** |
| **Disease duration** | 20.7 (15.5-28.9) | 21.2 (17.2-27.3) | 0.80 |
| **Time since last relapse, weeks** | 75.5 (30.5-146.0) | 69.0 (32.0-106.0) | 0.75 |
| **Disease-modifying therapy, n (%)** |  |  | 0.61 |
| Dimethyl fumarate | 5 (15.2%) | 4 (8.5%) |  |
| Interferon-beta | 6 (18.2%) | 7 (14.9%) |  |
| Glatiramer acetate | 2 (6.1%) | 1 (2.1%) |  |
| Natalizumab | 0 (0.0%) | 2 (4.3%) |  |
| Cladribine | 1 (3.0%) | 1 (2.1%) |  |
| Fingolimod | 3 (9.1%) | 2 (4.3%) |  |
| Alemtuzumab | 0 (0.0%) | 0 (0.0%) |  |
| None | 16 (48.5%) | 30 (63.8%) |  |
| **EDSS** | 6.0 (3.5-7.0) | 6.0 (4.0-6.5) | 0.70 |
| **NfL** | 10.9 (8.9-13.2) | 11.1 (8.7-14.2) | 0.65 |
| **Mean whole brain volume (L), SD** |  |  | 0.19 |
| **T2 lesion volume (mL)** |  |  | 0.66 |
| P values from χ2 test, Mann-Whitney test for median, and two sample t-test for mean. Data presented as n (%) or median (IQR), unless otherwise indicated. | | | |

| **Table S5. Demographics of the patients with PPMS** | | | |
| --- | --- | --- | --- |
|  | **MS (N=36)** | | |
|  | **Men (n=18)** | **Women (n=18)** | **p value** |
| **Disease duration** | 11.4 (5.3-18.3) | 5.9 (4.9-10.9) | 0.14 |
| **Time since last relapse, weeks** | 41.0 (35.5-65.5) | 31.5 (19.5-78.0) | 0.80 |
| **Disease-modifying therapy, n (%)** |  |  | <0.001 |
| Dimethyl fumarate | 0 (0.0%) | 1 (5.6%) |  |
| Interferon-beta | 5 (27.8%) | 1 (5.6%) |  |
| Glatiramer acetate | 0 (0.0%) | 0 (0.0%) |  |
| Natalizumab | 0 (0.0%) | 0 (0.0%) |  |
| Cladribine | 0 (0.0%) | 0 (0.0%) |  |
| Fingolimod | 0 (0.0%) | 0 (0.0%) |  |
| Alemtuzumab | 0 (0.0%) | 0 (0.0%) |  |
| None | 13 (72.2%) | 16 (88.9%) |  |
| **EDSS** | 4.0 (3.6-6.0) | 4.5 (3.5-6.0) | 0.94 |
| **NfL** | 9.7 (7.7-10.3) | 11.0 (9.0-14.1) | 0.10 |
| **Mean whole brain volume (L), SD** |  |  | 0.66 |
| **T2 lesion volume (mL)** |  |  | 0.81 |
| P values from χ2 test, Mann-Whitney test for median, and two sample t-test for mean. Data presented as n (%) or median (IQR), unless otherwise indicated. | | | |

**Figure S1.** **A)** Volcano plot showing the log2 fold difference of lipid mediator levels between relapsing-remitting MS (RRMS) and progressive MS (PMS) in men, and a similar plot (**B**) is shown for women. **C)** Volcano plot showing the log2 fold difference of lipid mediator levels between healthy control (HC) and MS in men, and a similar plot (**D**) is shown for women. Level of significance (p < 0.05) is indicated on the y-axis as –log10(p value), adjusted for multiple testing.

**Figure S2.** Spearman correlation analysis in the whole MS group, correlated with EDSS, NfL WBV, or LV, corrected for DMT use (yes/no), disease duration, BMI (only for NfL correlations), and MS subtype (RRMS/PMS).

**Figure S3.** Spearman correlation analysis in the men with MS group, correlated with EDSS, NfL, WBV or LV, and corrected for DMT use (yes/no), disease duration, BMI (only for NfL correlations), and MS subtype (RRMS/PMS).

**Figure S4.** Spearman correlation analysis in the women with MS group, correlated with EDSS, NfL WBV or LV, and corrected for DMT use (yes/no), disease duration, BMI (only for NfL correlations), and MS subtype (RRMS/PMS).
